# Multimodal Management of Supine Hypertension With Orthostatic Hypotension in an Elderly Male Patient With Parkinson’s Disease

**DOI:** 10.7759/cureus.82394

**Published:** 2025-04-16

**Authors:** Manik Dayal, Meet S Shah, Navid Radfar, Sagar Patel, Renjit Thomas

**Affiliations:** 1 Department of Internal Medicine, Rutgers University New Jersey Medical School, Newark, USA; 2 Department of Cardiology, Rutgers University New Jersey Medical School, Newark, USA; 3 Department of Medicine, Rutgers University New Jersey Medical School, Newark, USA; 4 Department of Cardiology, Veterans Affairs (VA) New Jersey Healthcare System, East Orange, USA

**Keywords:** autonomic nervous system dysfunction, continuous positive airway pressure (cpap), first-degree av block, multimodal management, neurogenic orthostatic hypotension, parkinson's disease, physiologic monitoring, recurrent syncope, supine hypertension, supine hypertension-orthostatic hypotension (sh-oh)

## Abstract

Supine hypertension with orthostatic hypotension (SH-OH) represents a paradoxical and challenging form of blood pressure (BP) dysregulation, particularly in patients with autonomic failure such as Parkinson’s disease (PD). This is a case of an 85-year-old male veteran with PD and multiple comorbidities, including coronary artery disease, diabetes with neuropathy, obstructive sleep apnea, and chronic kidney disease, who exhibited severe SH-OH characterized by supine systolic BP exceeding 200 mmHg and orthostatic systolic dropping to 110 mmHg. His symptoms included syncope, dizziness, and headaches. Pathophysiology involves autonomic dysfunction with impaired baroreflex, residual sympathetic activity, and dysregulation of the renin-angiotensin-aldosterone system.

Management was tailored to address both SH and OH. Nonpharmacologic strategies included head-of-bed elevation, fluid and salt supplementation, compression garments, and continuous positive airway pressure therapy, which also targeted his untreated OSA and helped reduce sympathetic overactivity. Pharmacologic interventions required fine-tuning due to the complex interplay of SH and OH. Fludrocortisone was contraindicated due to a recent upper gastrointestinal bleed. Pyridostigmine was trialed but discontinued after evidence of atrioventricular block. The final regimen involved clonidine and hydralazine at bedtime for SH, along with midodrine timed around daytime activity for OH.

This case highlights the nuanced, often counterbalancing management required in SH-OH, especially in elderly patients with neurodegenerative disease and cardiovascular risk. Multimodal therapy, individualized to avoid exacerbating one component while treating the other, remains essential. Further research is needed to optimize care strategies and improve the quality of life in this vulnerable population.

## Introduction

Supine hypertension with orthostatic hypotension (SH-OH) is a paradoxical autonomic dysfunction, commonly seen in neurodegenerative disorders such as Parkinson’s disease (PD), multiple system atrophy, and pure autonomic failure [1]. The incidence of SH with OH in the general population is not extensively documented in the literature; however, according to the Atherosclerosis Risk in Communities study, which included middle-aged adults aged 45-64 years, 4.4% of participants had OH. Among those with OH, 58% had SH [2]. SH-OH presents a clinical challenge, as SH exacerbates nocturnal end-organ damage while OH increases fall risk and morbidity [3]. The underlying pathophysiology involves impaired baroreflex sensitivity and autonomic failure, leading to inadequate norepinephrine release upon standing [4]. Additionally, medications already being taken by patients with PD, such as Levodopa, can further aggravate OH [1].

Diagnosis relies on postural blood pressure (BP) measurements; however, other confirmatory techniques can include tilt-table testing and plasma norepinephrine levels [4]. Treatment requires balancing both conditions. Nonpharmacologic strategies include fluid and salt intake, head-of-bed elevation, and compression garments, and now, continuous positive airway pressure (CPAP) therapy is emerging as a potential intervention for SH-OH, helping reduce SH and improve orthostatic tolerance [5]. Pharmacologic options range from midodrine and fludrocortisone to droxidopa and pyridostigmine [6-9] for OH and short-acting hypertensive agents for SH [10]. This case highlights the complexities of SH-OH management in patients with multiple comorbidities, illustrating the challenges of medication adjustments, patient adherence, and the need for individualized care.

## Case presentation

In July 2023, an 85-year-old male veteran with a history of coronary artery bypass grafting, PD, OH, diabetes mellitus with neuropathy, hypertension, hyperlipidemia, chronic kidney disease, and obstructive sleep apnea presented following three episodes of orthostatic syncope. He reported dizziness and headaches but denied chest pain, dyspnea, or visual disturbances. Although generally compliant with his medications, he occasionally missed doses due to financial limitations.

On admission, supine systolic blood pressure (SBP) exceeded 200 mmHg, with a sitting SBP of 130 mmHg and a standing SBP of 110 mmHg. His heart rate remained stable between 60 and 80 bpm across all positions. These findings confirmed the diagnosis of SH-OH. Initial management included nonpharmacologic strategies: compression stockings, elevation of the head of the bed to 30°, increased fluid and salt intake, and avoidance of abrupt postural changes. Pharmacologic interventions followed, starting with an increase in pyridostigmine from 30 to 60 mg daily, discontinuation of amlodipine, initiation of hydralazine as a short-acting alternative while lying, and the addition of clonidine at bedtime. Midodrine was prescribed for use before standing. These combined measures improved orthostatic symptoms and initial control of SH over a five-day period.

**Figure 1:**
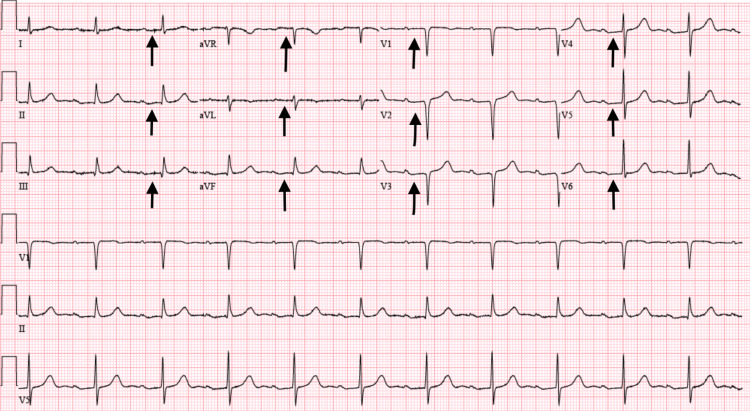
EKG showing the first-degree AV block after an increase in the pyridostigmine dosage. Black arrows point at PR intervals, which are greater than 0.2 seconds, supportive of the first-degree AV block AV: atrioventricular; aVR: augmented vector right; aVL: augmented vector left; aVF: augmented vector foot

The patient’s hospital course was complicated by anemia secondary to an upper gastrointestinal (GI) bleed. Endoscopy revealed healing duodenal ulcerations, and he was started on a proton pump inhibitor. Due to the recent bleed, fludrocortisone was contraindicated despite its known utility in managing OH. His PD was managed with carbidopa-levodopa 25/100 mg three times daily, which was recognized as a possible contributor to orthostatic symptoms but could not be adjusted due to the severity of his PD. Neurology recommendations included increasing pyridostigmine to 60 mg three times daily; however, the patient developed a first-degree atrioventricular block that progressed to a Mobitz type I block (Figure 1), leading to discontinuation of the medication.

CPAP therapy was considered to address both obstructive sleep apnea and SH-OH. Initially, the patient refused due to discomfort with the device. After allowing a brief trial, he reported symptomatic improvement, leading to plans for outpatient CPAP titration and education to promote long-term adherence. Hydralazine and clonidine were maintained for ongoing SH due to their short duration of action, minimizing the risk of worsening morning OH. Although not typically recommended in SH-OH, a short trial of losartan was attempted to reduce systolic BP below 180 mmHg. It was discontinued due to concerns about exacerbating OH the following morning.

During discharge, the patient’s BP had improved to 160 mmHg supine, 140 mmHg sitting, and 133 mmHg standing. Given his age, comorbidities, and elevated fall risk, a permissive hypertension strategy was pursued. Discharge planning included sleep medicine follow-up for CPAP titration, physical therapy for lower extremity strength and vascular tone, and social work support for medication access and assistance. Environmental modifications were also recommended to improve home safety and reduce fall risk.

## Discussion

PD often leads to hypotension due to dysautonomia, particularly from impaired sympathetic nervous system activity. Neurogenic OH arises from reduced norepinephrine release at the neurovascular junction, impairing vasoconstriction upon standing. Dopaminergic medications like levodopa can worsen OH through central sympathoinhibition, peripheral vasodilation, baroreflex dysfunction, and negative inotropic effects [1]. SH in patients with OH is linked to autonomic dysfunction, especially baroreflex impairment [11]. Residual sympathetic activity and denervation super sensitivity may increase supine BP, while loss of nocturnal dipping contributes to volume dysregulation [11]. Activation of the renin-angiotensin-aldosterone system (RAAS), despite low renin, can lead to elevated angiotensin II and aldosterone levels, stimulating mineralocorticoid receptors and raising BP [12]. According to the American Autonomic Society and the European Federation of Autonomic Societies, baroreflex impairment, RAAS disruption, and denervation super sensitivity are central to SH-OH pathogenesis [13].

SH-OH management requires separate strategies for each component. As noted from the study by Goldstein et al., Parkinson’s medications, especially levodopa, often aggravate OH, but complete discontinuation is impractical, and careful dosing adjustments are crucial [1].

Nonpharmacologic measures remain essential and are first line [7]. Strategies include increasing water and salt intake (guided by 24-hour urine sodium), head-of-bed elevation to reduce nocturnal diuresis, compression garments, and water boluses to stimulate adrenergic activity [6-11]. Biofeedback and countermaneuvers can also help improve standing tolerance. CPAP therapy has shown promise in treating both SH and daytime OH [5]. By raising intrathoracic pressure, CPAP reduces venous return and cardiac output, lowering supine BP dose-dependently and decreasing nocturnal diuresis. This preserves intravascular volume and improves daytime orthostatic tolerance [5]. However, adherence may be limited by mask discomfort, noise, and claustrophobia [14].

CPAP attenuates sympathetic nervous system activity, stabilizing BP and supporting autonomic regulation in SH-OH [5]. This effect is particularly important in SH-OH, as it addresses both extremes of BP dysregulation, reducing SH by lowering cardiac output and improving daytime OH by mitigating nocturnal diuresis and sympathetic overactivity [5]. This dual effect makes CPAP a compelling therapy in managing both extremes of BP dysregulation.

Pharmacologic treatments for neurogenic OH, although not nearly as effective as treating the SH-OH compared to nonpharmacological treatment, are almost often necessary and include fludrocortisone, midodrine, and pyridostigmine. These agents work best when timed before positional changes but may exacerbate SH. Fludrocortisone increases volume via sodium retention and catecholamine activity and is commonly combined with lifestyle changes like salt/fluid intake and compression garments [7,8]. However, it is contraindicated in patients with upper GI bleeding due to its potential for worsening GI complications [15]. Midodrine, an alpha-1 agonist, is well established for improving orthostatic tolerance [7]. Combining midodrine and pyridostigmine may provide better symptom control than pyridostigmine alone [7].

Pharmacologic management of SH typically involves short-acting antihypertensive agents administered at bedtime to selectively lower nocturnal BP without significantly affecting daytime levels [10]. Clonidine effectively reduces nighttime BP and nocturnal natriuresis, though it may cause residual morning hypotension [10]. Guanfacine serves as another short-acting option for managing SH [10]. Short-acting calcium channel blockers, such as isradipine or hydralazine, and short-acting beta-blockers like atenolol or metoprolol tartrate, can also be used at bedtime to control supine BP [10].

## Conclusions

This case illustrates the complexity of SH-OH management, requiring individualized, multidisciplinary strategies. Nonpharmacologic measures remain first line, medication adjustments must balance SH treatment without worsening OH. New research recommends the utilization of CPAP as the mainstay nonpharmacologic treatment measure. However, barriers to CPAP adherence highlight the need for patient-centered interventions, including education and practical adaptations. Future research should explore improvements in CPAP devices to improve compliance, as well as extensive pharmacological assistance and education on appropriate usage.
